# Annotations

**Published:** 1890-11-01

**Authors:** 


### ANNOTATIONS.

**London's Lunatics and Provision for Them.**

We are at present bringing out in the columns of The Hospital the third annual series of articles on the insane population of Great Britain, and our readers who have followed the "Story of the Insane from Year to Year" will have some idea of the admissions, discharges, and deaths taking place at our county and borough asylums. We have several times pointed out the admirable manner in which these asylums have been administered by our county magistrates, and almost the only adverse comment which could be made was, in some counties, the putting off too long the additions to the asylum fabric which the ever-increasing number of registered lunatics rendered imperative. While this delay was in all instances caused by a laudable reluctance on the part of the county magistrates to spend the ratepayers' money, there could not be the least doubt that the delay was an expensive matter for the local ratepayer, because provision had to be found for the patients, and this was done by transferring cases to the asylums of other counties, or, worse still, the patients were sent to private asylums. In county asylums, these out-county cases, as they are called, are charged at the rate of fourteen shillings a-week each, and in private asylums the guardians have to pay as high as nineteen shillings a-week, or perhaps more. In the meantime the average cost of maintenance in our county asylums is about eight and sixpence a-week, so that our readers may form some idea of the enormous sums which the ratepayers had to find owing to dilatoriness or reluctance on the part of those whose duty it was to provide the requisite accommodation. We have remarked in the articles named that all experience showed that those boards which did not anticipate the wants of their respective counties lagged behind these wants. The metropolis was no exception in this "provision by delay." On the contrary, the metropolitan boards were the worst offenders, doubtless because they had the greatest number of patients to look after, and it was rare to find that all the Middlesex lunatics were in their own asylums. At present they are found in many asylums—some so far away that it is well-nigh impossible for their friends to visit them, an additional grievance which those friends need not have placed on them. Hundreds of them are in the metropolitan licensed houses, and the payments made there are at least double what they could be maintained for in a county asylum. The Asylums' Committee are at present engaged in erecting a huge asylum at Claybury. The day of its opening will see it filled with London pauper lunatics, and, it is stated, that many will remain boarded out in other institutions. The Asylums Committee will, therefore, it is much to be desired, at once take the preliminary steps for building the necessary accommodation, and will push on with the work, doing their best to keep well abreast of the demands on them. This accommodation should be at least equal to two per thousand of the whole population of the district, and as the metropolis is growing rapidly, it should be true economy to make it considerably more than that. It is hinted that the friends of many of the patients could afford to keep them as private patients, and that by looking out these cases the necessity for further building might be postponed for a time. The new Lunacy Act wisely defines a pauper lunatic as one who is in receipt of relief, or who needs relief for his proper treatment, and it is highly probable that the vast majority of the patients in the London asylums come under either of these conditions. A private patient cannot be maintained in a private asylum for less than twenty or twenty-five shillings a-week; or in a county asylum for less than fifteen or sixteen shillings. Manifestly, the number of patients whose friends could pay even the lowest of these sums is so small that it would not affect the question at all; and, if they did exist in sufficient number, one would be inclined to ask, "Where are they to find private asylums at which they could be admitted?" By sending their paupers to the Metropolitan licensed houses the London authorities have closed the doors against those cases whose friends could pay reasonable sums for their maintenance. Let the London Council at once withdraw their seven or eight hundred lunatics from these institutions, and we should probably see their proprietors admitting private patients at low rates. While regretting the necessity for building more asylums, we feel sure the Asylums' Committee are pursuing an enlightened policy in the present instance. One word of advice we will venture on. In no case should the asylum exceed one thousand beds; and whether one or more than one be needed, let it or them be managed exactly on the model of our provincial asylums and let the old metropolitan system pass away for ever.

**Science Broadens Slowly Down.**

"Freedom broadens slowly down, from precedent to precedent," sings Tennyson. That is perhaps altogether as it should be. But there is no reason whatever why certain elementary principles and facts of science, which are as easy to understand as the first four rules of arithmetic, and as necessary to the well-being of the public as food is to a hungry man—there is no reason at all why these facts and principles should "broaden slowly down," but every reason why public authorities and private individuals should seize upon them promptly, and apply them thoroughly in every case where necessity demands. The daily papers have told us a melancholy and discreditable story about the parish of Cheam in Surrey. This parish is almost within the sound of Bow bells; and yet the Rev. J. C. Sparrow, of St. Phillip's Parsonage, Worcester Park, makes himself responsible for the following story. Writing to the rural sanitary authority at Kingston for the use of the Kingston Isolation Hospital, Mr. Sparrow stated that they were visited with diphtheria in the parish, and that it had assumed a malignant form, several deaths having occurred. A great number of cottages in the parish were unfit for human habitation, and there were no means whatever of isolating the persons attacked. The sick and the well, the living and the dead, were hopelessly huddled together under the same roof. One family lived in a dirty, badly-built cottage, having one living-room downstairs, and two compartments, he could not call them rooms, upstairs. A boy in the house was taken ill with diphtheria and died. Within a few days the father took it and died, whilst two other sons were down with the disease in the next apartment. One of these brothers died a few days afterwards, and his death struggles were witnessed by the surviving brother, who shared the bed with him. Mr. Sparrow related other facts of a like nature, and concluded his letter to the authorities by an urgent appeal for help. Cheam, it appears, is in the Epsom sanitary district. Epsom was accordingly applied to for the means of isolation, but could do nothing, presumably because it has no isolation hospital. Kingston, which possesses such a hospital, was then appealed to as a last resort. But, because Cheam was not in the Kingston district, the hospital was, it is stated, reluctantly refused. Now both these districts are under the Local Government Board. Was it not possible that the cases of diphtheria occurring at Cheam might have been received at Kingston in the absence of any provision at Epsom, and the cost charged to the Epsom authorities? It is a disgrace to the common sense and right feeling of Englishmen that they should be so incompetent and so red-tape-bound when important crises of this kind come upon them. Whilst officials are wrangling and shaking their heads, and uttering their childish non-possumus, malignant diseases are spreading far and wide, and whole parishes are threatened with decimation. Epsom, we admit, should have an Isolation Hospital of its own, and so should many other towns of like wealth and importance. But as yet they do not possess such institutions. Until they can be forced by public opinion or statute to erect them, the Local Government Board should attach every parish and hamlet in the Kingdom to the nearest Isolation Hospital, and charge expenses which may occur to the parishes using the hospital. It is as stupid as it is scandalous that infectious diseases should spread throughout the land merely because officialism has not the capacity or the good will to make the best use of existing means of isolation.

